# Supplementary material for: Are carbon emissions trading and green financial instruments synergistic? -Comprehensive quantitative research based on content analysis
Source: PLoS One. 2024 Mar 7;19(3):e0298601. doi: 10.1371/journal.pone.0298601 (PMC10919601; doi:10.1371/journal.pone.0298601)
Supplement: S1 Data — (ZIP) [file pone.0298601.s001.zip › Data/地级市Z_F工作报告.zip!/╜¡╦╒/╤∩╓▌╩╨/└┤╫╘╣1⁄2╓┌║┼í╛┬φ┐╦╩2╛▌═°í┐_2018.docx]

各位代表:

现在,我代表扬州市人民政府向大会报告工作,请予审议,并请市政协委员和其他列席人员提出意见。

2017年工作回顾

2017年是党的十九大胜利召开的喜庆之年,是本届政府履职的开局之年。我们在中共扬州市委正确领导下,深入贯彻新发展理念,紧扣“两聚一高”,办好“十件大事”,统筹做好改革发展稳定工作,全面完成年度各项目标任务。预计实现地区生产总值5100亿元,增长8%;一般公共预算收入320亿元;全社会固定资产投资增长12%;城乡居民人均可支配收入分别增长8.4%、8.8%。

1、深化供给侧结构性改革,实体经济平稳向好。

促进转型升级,制造业质态不断提升。实施智能制造及高端装备等产业发展专项政策。预计全年规上工业增加值、开票销售、入库税收分别增长8%、18%、7%。工业投资2008亿元,占固定资产投资比重提升到55%。高新技术产业产值占规上工业比重达45%。战略性新兴产业增加值占GDP比重达16.5%。完善工业重大项目“四新”认定考核体系,全年新签约工业重大项目65项、新开工51项、新竣工102项、新达产77项。净增规上工业企业123家。百强企业开票销售增长面提升14个百分点。新增省示范智能车间14个、工业机器人384台套。获批国家智能制造试点示范项目3项。扬州高新区数控成形机床产业成为国家创新型产业集群试点。全年压减钢铁产能88万吨、水泥90万吨、造船290万载重吨,降低企业成本86亿元。

强化引领带动,现代服务业快速发展。制定现代服务业提质增效三年行动计划。服务业增加值占GDP比重提升1个百分点。软件和互联网业务收入1095亿元,增长30.2%。实施服务游客建设宜游城市“3号文件”,过夜游客增长16%,旅游业总收入780亿元。马可波罗花世界、汉陵苑创成4A级景区。新增4A级物流企业1家、3A级3家。新引进银行、保险、证券机构10家,新增境内外上市公司4家、“新三板”挂牌公司15家。新增信贷投放480亿元、直接融资259亿元。科技服务业业务收入增长12%。净增文化列统企业70家。电商交易额770亿元,增长40%。全年社会消费品零售总额增长10.1%。

注重优质高效,农业现代化取得实效。支持返乡下乡人员创业创新,促进农村一二三产业融合发展,19个农业园区入选全国农业创业创新园区目录。粮食生产再获丰收,全年粮食总产达289.3万吨。新增粮食仓容10万吨。新建高标准农田20.5万亩,划定373万亩永久基本农田。新增设施农(渔)业11.3万亩。新实施国家级农业科技重大专项2项。新创省级示范家庭农场25家。新增省级农产品品牌45个。农业机械化水平达86%。顺利完成第三次全国农业普查任务。供销、气象等工作得到加强。

推进多元合作,对外开放步伐加快。组织开展“烟花三月”国际经贸旅游节集中招商、赴沪双月招商、百日招商以及“名企名城行”系列招商活动,签约项目272个。稳步推进与中船重工、中航工业、中电科等央企合作,新落户世界500强及跨国公司项目6个,宝应生态渔业光伏发电“领跑者”示范基地、瘦西湖景区华侨城文旅项目、江都光线(扬州)中国电影世界等投资100亿元以上重特大项目实现突破。全年实际利用外资12.05亿美元。鼓励企业“走出去”,实现出口75亿美元,外经营业额增长10%。支持建筑企业拓展境内外市场,全年建筑业产值3550亿元,增长6%,4家企业获批特级资质,2个项目获鲁班奖。成功举办世界地理标志大会、上合组织国家协调员理事会会议、世界运河城市论坛、世界体育赛事与旅游峰会。

2、大力实施创新驱动战略,发展活力持续增强。

突出科技支撑,创新成效加快显现。持续推进“12345”创新发展工程,国家创新型试点城市通过验收。全社会研发投入占GDP比重提升至2.4%。新获批国家高新技术企业110家,新开发省级高新技术产品712件。新增省级以上“三站三中心”123家。新引进知名高校研创中心56家。发明专利授权量突破900件。新增国家、行业和地方标准30项。扬州高新区创成国家知识产权试点园区。玉器产品质量监督检验中心通过国家验收。“扬州漆器”创成国家地理标志商标。构建“2+N”人才政策体系,451人入选省级以上重点人才引进培养计划,柔性引进省“科技副总”133人。

做优载体平台,集聚效应逐步形成。出台推动全市开发园区转型升级系列政策和考核办法。新获批国家特色产业基地2个、省级科技产业园区3家。促进省级以上开发区承接上海、苏南园区亿元以上项目31个,总投资174.8亿元。全市新建科技产业综合体89万平方米,累计入驻企业1700家。引进优客工场、草根、酷立方等知名孵化器,新增国家级孵化器1家、众创空间6家,省级众创社区2家、众创空间12家,累计吸引超1000家企业、3000名创客入驻。

加大扶持力度,小微企业蓬勃发展。推进全国小微企业“双创”示范建设,搭建“1+N”网络服务平台,建成创业示范基地47个。新认定市级小企业基地13家。发放小微企业服务券、创新券2亿元,对小微企业厂房租赁、购买服务等给予奖补7000万元。优化融资服务,“e融扬州”资金池规模达21亿元,全年小微企业贷款余额1476.8亿元,增长11.7%。对小微企业引进本科以上大学毕业生给予1年社会保险补贴。全市小微企业营业收入4760亿元,增长41.4%。

3、彰显生态宜居特色,城乡面貌日益改善。

完善功能布局,城市品质进一步提升。推进宁镇扬一体化, S611沿湖大道、宿扬高速建成通车,连淮扬镇铁路扬州段、城市南部快速通道、金湾路等快速推进,五峰山过江通道公路接线、扬州泰州国际机场一期扩建、真州路北延、扬子津大桥等开工建设。淮河入江水道整治基本完成。长江堤防防洪能力提升工程、瓜洲外排泵站工程加快实施。深入推进公园体系建设,全市新建改造8个综合公园、81个社区公园、29个口袋公园,三湾公园、蜀冈文化公园建成开放,明月湖体育休闲公园提档升级。启动东南片区更新改造,推进南河下街区整治。市区新辟调整公交线路21条,出新城市家具577处;新建污水管网160公里、污水集中处理率90%;建成生活垃圾分类试点社区10个、示范小区36个。

推动城乡统筹,乡村环境明显改变。加快首批10个市级特色小镇建设,头桥镇入选省级特色小镇创建单位,杭集镇获批全国特色小镇,仪征枣林湾入选国家首批运动休闲特色小镇试点。启动实施特色田园乡村“111”行动,沙头镇沙头村和月塘镇四庄村入选省级试点,方巷镇沿湖村获评国家级“最美渔村”。建成51个省级绿化示范村。新改建农村公路180公里、农桥50座。镇村公交开通率84%。执行《扬州市河道管理条例》,全市四级河长体系全面建立。清淤县乡河道740万方、村庄河塘970万方,创成省级水美乡镇4个、水美村庄21个。

厚植绿色根基,生态文明建设富有成效。扎实推进“263”专项行动,关闭搬迁禁养区内畜禽养殖场844家,超额完成省定32万吨减煤任务,关停化工企业103家,砖瓦行业专项整治取得进展,完成市区27条、县(市)9条黑臭水体整治。江淮生态大走廊建设纳入国家《长江经济带生态环境保护规划》,27项年度重点工程扎实推进,制定宝应、高邮江淮生态经济区建设“四个清单”。圆满完成省环保督察迎检任务,全部落实环保信访交办件。完成大气“国十条”考核目标,PM2.5年均浓度较2013年下降22.9%。创成国家水生态文明城市。32个省考以上断面水质达标率93.8%。仪征滨江水源地取水口整治到位。凤凰岛创成国家湿地公园。顺利通过国家节水型城市、国家森林城市复查。全市新增植树569万株、成片造林3.05万亩、永久性保护绿地4块。新增绿色建筑面积191万平方米。扬州环保产业园成为国家循环经济标准化试点园区。广陵获得全国首批国土资源节约集约“四个创新”示范点称号。

4、着力保障和改善民生,不断增进人民福祉。

立足普惠均衡,社会事业协调发展。市八届人大一次会议确定的食品安全、环境保护、社区建设等民生实事全面完成。全市新增就业创业22.4万人次、农村劳动力转移就业1.1万人。深入推进精准扶贫、阳光扶贫,全市建档立卡低收入农户基本达到人均可支配收入7000元,沿江、沿河地区经济薄弱村集体经济年收入分别达50万元、40万元。新改扩建幼儿园11所,建成中小学7所。资助在校困境儿童1200人,新招收中小学宏志班26个。新创省义务教育现代化学校51所。高考再次取得佳绩。建成江苏旅游职业学院和扬大广陵学院新校区。龙虬庄遗址入选国家考古遗址公园立项名单。木偶研究所被联合国教科文组织授予中国木偶艺术人才传承培训基地。新设24小时城市书房11家。入选省首批书香城市建设先进市。扬州广播电视台获评全国年度最具综合实力城市台。整合城乡居民基本医疗保险制度,跨省异地就医实现联网结算。18家农村区域性医疗卫生中心全面建成使用,115家基层医疗卫生机构全部参与医联体。迁建宝应、江都人民医院,新建高邮人民医院东院。积极开展省运会筹备工作,建成游泳健身中心、射击运动中心。成功举办鉴真国际半程马拉松赛、女子佩剑世界杯赛、深潜赛艇海上丝路公开赛。世园会、省园博会各项准备工作有序推进。台湾事务、侨务、科普、档案和工会、青年、妇女等工作取得新进步。

彰显公平正义,社会治理有效加强。全国文明城市创建实现“三连冠”。全省城市治理与服务现场会在扬召开。江都荣获全国社会治安综合治理最高奖“长安杯”。党的十九大安保维稳任务圆满完成,全市社会大局持续稳定。建成市级公共法律服务中心。组建市反通讯网络诈骗中心,冻结止付被骗资金9092万元。全市安全生产事故起数和死亡人数分别下降46%、32.9%。食品抽检合格率97.8%。宝应获评全省首批食品安全示范县。实施社区建设行动计划,在61个村开展社区建设试点,在29个城区社区推行全科社工。加强国防教育,推进国防动员和后备力量建设,支持扬州舰、高邮湖舰赴亚丁湾海域护航并出访西欧四国。民族、宗教、仲裁、应急等工作取得积极成效。

5、加强政府自身建设,服务效能不断提升。

能力作风持续强化。深入学习贯彻党的十九大精神,自觉用习近平新时代中国特色社会主义思想武装头脑、指导实践、推动工作。常态化制度化推进“两学一做”学习教育,广泛开展“聚焦富民·走千村访万户”大走访大排查活动。召开市政府全体(扩大)会议专题推进能力作风建设。开展落实中央“八项规定”精神和纠正“四风”情况专项检查。严格执行“三直接”环节操作规范。法治政府、廉洁政府建设取得新成效。

政务公开持续推进。启动“云上扬州”建设。创办《扬州市人民政府公报》。办理依申请公开答复1023件。公开重点领域、重要事项政府信息25万条。召开新闻发布会260场。推进财政预决算和“三公”经费公开。12345热线和《寄语市长》回复满意率98%以上。综合信用指数在全国保持前十。

行政效能持续提升。深化“放管服”改革,“不见面审批”覆盖市县行政审批业务90%以上,“双随机一公开”实现所有领域所有事项全覆盖。推动政务服务“一张网”建设,“扬州旗舰店”上线运行。推进“3550”改革,在全省率先推行“十二证合一”“九证照联办”,不动产登记实现“一窗受理、集成服务”,施工许可平均审批时限缩短到48.5个工作日。组建市开发区、江都开发区行政审批局。办理市人大代表议案、建议325件,政协提案544件。

各位代表,成绩来之不易,这是市委正确领导的结果,是市人大、市政协和社会各界大力支持的结果。在此,我谨代表市政府向全市人民,向市人大代表、政协委员,向各民主党派、工商联、无党派人士、各人民团体、驻扬各单位,向所有关心支持参与扬州建设的朋友们,表示崇高敬意和衷心感谢!

在肯定成绩的同时,我们也清醒地看到存在的问题,主要是:经济转型升级步伐不快,做大总量与提升质量的双重任务仍然艰巨;创新发展动力不足,企业研发投入不够,科技产业综合体“建管用”水平有待提高;城乡、沿江沿河发展不平衡不充分的问题依然存在,县域经济整体实力还需增强;环保问题仍然突出,生态文明建设任重道远;金融领域可能引发的社会风险依然存在;民生工作与群众对美好生活的需要还有差距;一些部门敢于担责、善于担当的精神和能力不足,等等。对此,我们必须高度重视,采取有力措施加以解决。

2018年工作安排

2018年是全面贯彻党的十九大精神的起始之年,是实施“十三五”规划承上启下的关键一年。政府工作的总体要求是:全面贯彻落实党的十九大精神,以习近平新时代中国特色社会主义思想为指导,按照习总书记视察江苏重要讲话要求和中央经济工作会议、省委省政府工作部署,坚持稳中求进工作总基调和新发展理念,深刻把握转向高质量发展的根本要求,紧扣“两聚一高”,办好“十件大事”,统筹做好稳增长、促改革、调结构、惠民生、防风险各项工作,团结带领全市人民共同创造美好生活,把“强富美高”新扬州建设不断推向前进。

今年政府工作的主要目标是:

——经济支撑型指标夯实巩固,切实坚持“稳中求进”。GDP增长8%左右。规上工业增加值增长8%左右。一般公共预算收入增长6%。全社会固定资产投资增长11-12%,其中工业投资占比超过55%。社会消费品零售总额增长10%。实际利用外资12亿美元。出口实现正增长。

——质量结构型指标调高调优,持续聚焦创新发展。全社会研发投入占GDP比重提升至2.5%、万人发明专利拥有量达11件。工业技改投入、开票销售、入库税收分别增长15%、8%、7%。高新技术产业产值占规上工业比重提升1个百分点。战略性新兴产业增加值占 GDP比重达 17%。服务业增加值占GDP比重、生产性服务业占服务业比重均提升1个百分点。种植业“三品”比重达53%。全年新增市场主体5万个。

——生态约束型指标压紧压实,加快打造秀美扬州。市区空气质量优良率70%。清洁能源占比提升1个百分点。省考以上断面水质优良比例65.6%以上,饮用水源水质达标率保持100%。林木覆盖率达到23%。自然湿地保护率48%以上。主要污染物排放、单位GDP二氧化碳排放强度下降、单位GDP能耗下降完成省定目标。

——文化发展型指标不断完善,力促文化繁荣兴盛。推动大运河文化带扬州段成为显著文化标识。巩固提升文明城市创建成果,城市文明程度指数位居全国前列。新设24小时城市书房10家。开展公益性文化活动200场次以上。新建村(社区)综合文化服务中心312家。新增文化列统企业50家。

——民生幸福型指标丰富内涵,倾力创造美好生活。采集就业岗位9万个,城镇新增就业创业20万人次以上。城镇、农村常住居民人均可支配收入均增长8%以上。居民消费价格指数和城镇登记失业率不高于省定目标。常住人口城镇化率达67%。新建提升各类公园65个。体育人口比例达39%。加强诚信体系建设,切实维护公平正义。

围绕实现上述目标,按照市委七届五次全会要求,重点抓好以下六方面工作:

1、聚焦供给侧结构性改革,持续壮大实体经济,在经济平稳健康发展上转向高质量。

强化项目支撑,筑牢转型升级基础。围绕推进“项目质量优化年”,做到“四个提高”:提高项目招引组织程度。常态化开展“6+X”招商引资活动,紧盯大型央企国企、百强民企和世界500强企业,瞄准龙头带动型、科技创新型、重大技改型项目,全面推进基本产业和特色产业补链扩链强链合作。全年新开工重大工业、服务业、农业项目50个、40个、30个以上,推动重特大产业项目实现突破。提高项目质量。以高质量项目引领高质量发展,新开工项目中新建项目比重不低于50%,基本产业和先进制造业项目、重大智能化项目比重分别达60%和30%,实施重点技改项目200项以上。提高项目投入产出效益。围绕亩均投资、销售、税收和竣工后研发投入等指标开展重大项目绩效评价。提高项目督查考核实效。每月召开一次工业重大项目过堂会,每季度组织一次集中观摩。通过开工认定的重大项目两年内实现竣工率、达产率、列统率3个“百分百”。

强化企业支撑,提升存量经济质态。鼓励企业家深耕实体经济,加大研发投入,全力以赴增品种、提品质、创品牌,定期开展企业家论坛和“创二代”领航论坛,营造支持、尊重企业家的社会氛围。实施强企工程和专精特新、小巨人企业培育计划,全市净增工业规上企业100家以上。遴选培育100家以上重点骨干企业,推进打造行业领军企业。实施创新型企业“215”培育工程、企业技术中心建设“双百”计划。以效益增长、资产增值为核心,支持国有企业做强做优做大。激发民营企业投资活力,民间投资占固定资产投资比重70%以上。提升民营企业发展质态,推动个转企1600户以上、规改股30户以上。鼓励优质企业加快进入多层次资本市场,建立80家上市、挂牌后备企业库。发放小微企业服务券、创新券各1亿元,小微企业营业收入达5200亿元。

强化产业支撑,打造现代化产业集群。一是加快建设“制造强市”。重点实施“四大计划”:智能制造和高端装备提升计划。推进江苏微机电产业园和研究院、哈工大机器人科创中心及扬州微电子产业园建设,打造高端先进制造示范园区。全年新建智能车间20个以上,工业机器人及智能装备保有量3500台套以上,培育服务型制造示范企业10家,招引10个以上高端装备项目,创新研制10项以上具有业内领先水平的首台套重大装备及关键零部件。高端装备产业产值增长10%。汽车产业强链补链计划。加快新能源汽车、汽车后市场、新型动力电池等项目引进,开工建设仪征上汽大众整车二期工程、市开发区中集通华数字化半挂车产研联合体,全年实现汽车产业产值1100亿元。战略性新兴产业和军民融合产业强基计划。重点促进新能源新光源、新一代信息技术、生物医药等产业壮大规模,战略性新兴产业产值增长12%。加快中船重工高邮试验场、中电科仪征试验场等军民融合产业园区建设,争创首批省级军民融合创新示范区。传统特色产业升级计划。推动中海工业大型集装箱船和中航鼎衡高技术化学品船扩大制造规模,大力发展电子化学品等高附加值中间体和精细化工产品。推动食品、服装、日化、工艺美术等产业放大品牌效应、做大市场规模。二是加快提升服务业发展水平。重点推进“四大工程”:现代服务业提质增效工程。完善专项扶持政策,全力发展软件与互联网产业,加快“云上扬州”建设,推动大数据、云计算、物联网等行业项目落户,打造新兴软件名城,全年业务收入增长30%。继续推进以“两古一湖”为代表的文化休闲旅游,推动瘦西湖创建国家级旅游度假区,开工建设华侨城文旅项目,规划建设长江国际客运码头。创建全国旅游标准化试点城市,全面提升宝应、高邮和仪征县域旅游发展水平。全年接待过夜游客超过820万人次,旅游业增加值占GDP比重达7.8%。增强金融服务实体经济能力,实现直接融资200亿元以上。集聚区转型发展工程。提升11个省级以上现代服务业集聚区建设水平,全年营业收入1300亿元、税收36亿元。推动国庆路、“双东”街区创建省级老字号集聚示范区。重点企业培育工程。实施服务业重大项目“四新”认定办法,全年净增重点服务业企业100家。质量提升和融合发展工程。完成5项服务业标准制定,争创国家级传统工艺产业标准化建设示范基地。培育电子商务领军企业,电商交易额增长30%以上。三是加快推进农业现代化。重点打造“四大板块”:生态农业发展板块。推动10个农业园区争创国家和省农业产业示范园。新建高标准农田15万亩、粮食仓容10万吨以上,切实保障粮食安全。高效经济作物种植板块。建设300万亩优质粮油种植基地、100万亩绿色园艺种植基地。优质畜禽水产养殖板块。打造5个现代化生猪产业集聚区,建设3000万羽特色家禽饲养基地、95万亩特色水产养殖基地。新兴业态发展板块。观光休闲农业年营业收入增长20%,农村电商企业数、销售额分别达390家、50亿元;培育村经济合作社10个、省级示范家庭农场20个。

推动三大变革,着力提高全要素生产率。一是突出质量变革。打响“扬州制造”“扬州服务”质量品牌,新增省级以上品牌17个、地理标志商标2件、商标注册4000件,发明专利授权量突破1000件,制(修)订国际、国家、行业和地方标准20个以上。实施淘汰落后产能专项行动,全面完成省定节能、减排、减化目标。二是推进效率变革。提升园区承载效率。围绕“一特三提升”推动园区转型、集约、绿色、融合发展,市开发区、扬州高新区分别列全省同类园区前10位、15位,其他园区进位2位以上。提升要素配置效率。全年银行机构新增贷款400亿元以上,制造业贷款占全部贷款比重不低于15%。完善土地保供体系,保障高质量项目建设用地需求。全年为企业减负100亿元以上。三是深化动力变革。发挥创新引领作用。大力发展以实验室为载体的科技研发产业,在智能制造、软件信息、军民融合、生猪选育等领域着力引进一批重点实验室。完成全国小微企业“双创”示范三年行动计划。启动新一轮创新型城市建设,全年开发省级高新技术产品500件以上,促成产学研合作项目430个以上。新建科技产业综合体和众创空间120万平方米,新入驻企业1000家,创成省级以上科技企业孵化器3家。科技服务业业务收入突破100亿元。深入实施“绿扬金凤计划”“英才培育计划”,全面落实市“人才政策20条”,全市人才总量达81万人。增强消费拉动作用。积极创造优质消费产品,满足多样化、个性化消费需求,社会消费品零售总额达1645亿元。强化开放驱动作用。组织企业参加10场以上“一带一路”推介会,推动高新技术产品出口占比提高1个百分点。全年新落户“530”项目6个、新增“510”项目2个;实现出口额76亿美元、外经营业额增长5%。

2、推进城市高品质建设,加快乡村振兴步伐,在促进城乡协调发展上转向高质量。

全面对接国省战略,推进重大基础设施建设。主动融入“一带一路”、长江经济带和省“1+3”重点功能区战略,推动仪征先导区与南京江北新区、六合区全面衔接,加快推进连淮扬镇铁路、东部综合交通枢纽、五峰山过江通道公路接线等重点工程,北沿江高铁、润扬第二过江通道、宁扬城际、龙潭过江通道等项目前期工作力争取得突破性进展。扬州泰州国际机场一期扩建工程建成投运。继续推进长江镇扬河段三期整治、长江堤防防洪能力提升、瓜洲外排泵站和通扬线航道整治等工程。加强长江岸线动态管理,优化岸线资源综合利用。做好新一轮城市总体规划修编。

突出内涵品质提升,做优做美中心城市。深化公园体系建设,推动新城“公园+”、老城“+公园”。贯彻《扬州市公园条例》,建立分级分类管理机制。推进城市“双修”试点建设,突出抓好东南片区更新改造、江广融合区生态修复和历史城区城市修补。完成城区41个主要道路交叉口园艺化改造。落实《扬州古城保护条例》,实施南通路–南河下街区综合整治。有序推进生态科技新城、广陵新城、西区新城建设。进一步完善城市路网体系,江广高速公路改扩建、沪陕高速枣林湾互通、城市南部快速通道、金湾路、S352江都段建成通车。开工建设G345仪征新集至刘集段、G328江都段、扬子津路,启动润扬路、运河路、江平路快速化改造。大力疏解交通堵点,完成真州路北延、杨柳青路西延、平山堂路西延工程。新铺设污水管网48公里。深化国家公交都市和省公交优先示范市建设,结合城郊公共交通发展,新辟调整公交线路12条,新增新能源或清洁能源公交车302辆、占比达85%。实施垃圾分类和治理专项行动,启动市区生活垃圾焚烧发电厂三期、赵庄垃圾卫生填埋场二期建设。加强网约车、共享单车、无人机、寄递物流、群租房等业态监管。推进物业管理标准化、规范化,建立居民满意度监督评价机制。

推进城乡深度融合,加快乡村振兴步伐。按照“产业兴旺、生态宜居、乡风文明、治理有效、生活富裕”的总要求,科学制定“乡村振兴行动计划”。坚持质量兴农、绿色兴农,落实农业供给侧结构性改革举措。推进“一园一区三基地”建设,打造以宝应、高邮为重点的生态农业发展样板区。调整优化乡镇工业集中区布局,支持县域打造一批生态型、创新型、专业化园区。突出产业引领,新增省级特色小镇创建对象2个。推进特色田园乡村试点建设。深入推行“河长制”,疏浚整治县乡河道50条、村庄河塘200条,新创成1个省级水美乡镇、10个省级水美村庄。按照基层基本公共服务功能配置标准,均衡配置城乡公共资源。全市新增农村劳动力转移就业1万人,加快农业转移人口市民化。培育新型职业农民1.5万人。推进县乡农村产权交易市场规范化建设,全年交易额14亿元以上。推进精准扶贫,实施“三保五助”扶贫开发工程,规范运行“阳光扶贫”监管系统。沿江、沿河地区80%的经济薄弱村集体经济年收入分别达55万元、45万元。继续做好村级债务化解工作。健全自治、法治、德治相结合的乡村治理体系。

3、坚持人与自然和谐共生,推动形成绿色发展方式和生活方式,在生态文明建设上转向高质量。

坚持更高标准,着力解决突出环境问题。全面落实中央、省环保督察整改意见,深入开展“263”专项行动。坚持“五气”同治,淘汰替换35蒸吨及以下燃煤锅炉6台,综合治理挥发性有机物排放企业107家,渣土运输车全部密闭化改造,落实秸秆禁烧和综合利用措施,市区全面禁放烟花爆竹,PM2.5年均浓度较2015年下降10%。完成赵家沟、大汪河等52条河道综合整治,加快七里河、槐泗河等8条河道整治进度,基本消除城市黑臭水体。新改建宝应柳堡、高邮珠光、江都晴川等污水处理厂,新增150个行政村污水处理设施。各县(市、区)30%以上乡镇污水处理设施保证正常运转,省考以上断面沿线乡镇污水处理设施全部投入使用,消除劣V类水体。加强土壤污染监测,设置240个农产品产地污染防治监控点。畜禽规模养殖场治理率和粪便综合利用率分别达70%、96%。

强化系统思维,加快构建生态廊道。实施扬州市主体功能区规划,守住生态保护红线。完善江淮生态大走廊建设规划。加快高宝邵伯湖“三退三还”工程,退渔还湖面积2万亩以上。实施生态体育休闲公园的园林化、园艺化改造,加快建设“三河六岸”公园先导区、三江营湿地公园。推进宝应湖、枣林湾等十大生态中心建设,扩建三湾生态中心。新增植树660万株、成片造林3万亩,恢复湿地4000亩。市区新增绿地100万平方米。

推进绿色发展,用心守护绿水青山。加快国家循环经济示范城市建设,实施市级以上开发园区生态化、循环化、低碳化改造。强化新能源政策引导,推动宝应生态渔业光伏发电、高邮多能互补集成等示范项目建设。推动40家企业列入清洁生产审核计划。新增二星级以上绿色建筑80万平方米,建筑业产值增长5%。新增汽车充电站40座、充电桩500个。深入开展节约型机关、绿色家庭、绿色学校、绿色社区创建行动,倡导简约适度、绿色低碳的生活方式。

4、进一步坚定文化自信,弘扬优秀传统文化和地方特色文化,在推动文化发展上转向高质量。

打造文化高地,高标准推进大运河文化带建设。围绕省委提出的“打造高颜值的生态长廊、高品位的文化长廊和高效益的经济长廊”要求,编制《大运河文化带扬州段建设规划》。加强运河沿岸环境综合整治和生态修复,严格落实宝应、高邮江淮生态经济区建设“四个清单”。挖掘运河文化内涵,强化遗产保护管理,启动建设中国大运河博物馆等“一馆多园”。加快瓜洲、湾头、邵伯等古镇保护开发,整合大运河旅游发展资源,培植壮大文化旅游、现代物流、观光休闲农业等沿河特色产业。支持WCCO办好省级层面的运河城市论坛。

促进文化繁荣,擦亮文化名城品牌。出台推进现代公共文化服务体系建设实施意见,强化基层文化团队建设,提升书香城市建设水平。实施“名城名人名作”工程,推进“文化名师工作室”建设,创作扬剧、木偶剧、曲艺等一批精品力作。举办朱自清先生诞辰120周年纪念活动。承办第十届中国曲艺牡丹奖颁奖系列活动。建设隋炀帝墓考古遗址公园。大力发展文化创意产业,办好文化产业博览会和文化旅游产品创意设计大赛,推动历史文化资源活化转化。加快江都光线(扬州)中国电影世界等文化产业项目建设,启动实施琴筝文化产业园,规划建设玉文化产业园。

坚持城乡共建,提升文明城市建设内涵。市县联动,实施文明城市创建新三年计划,将扬州高新区等6个园区纳入常态长效建设月度考评。积极开展各类评先评优活动,培育和推选一批道德模范、扬州好人及最美人物。大力推进诚信体系建设,探索将不文明行为纳入公民个人征信系统。加强社会心理服务体系建设,培育自尊自信、理性平和、积极向上的社会心态。

当好东道主、展示新形象,精心承办好两大盛会。做好第十九届省运会和第十届省园博会各项承办工作,努力办成展示名城形象、赢得群众口碑、留下深远影响的里程碑赛事和展会。开展“三路一环”和城市主要出入口、比赛场馆周边、宾馆酒店周边安全大整治、交通大整治、环境大整治,确保以崭新面貌迎接八方宾客。建成广陵体操馆、南部体育公园和省园博会主展馆、锦云村、园冶园等工程。扎实抓好训练备战工作,争取参赛总成绩实现最好目标。深入实施省运惠民“六边工程”,实现全民健身中心乡镇全覆盖。做好省园博会与世园会的有机衔接,完成世园会总体规划方案。

5、不折不扣落实好各项富民惠民举措,切实增强群众获得感幸福感安全感,在保障和改善民生上转向高质量。

优先发展教育事业,提升群众健康水平。推进教育现代化工程。新改扩建公办幼儿园9所,新建中小学7所。实施《扬州市关于进一步加强普通高中教育的若干意见》。支持职业教育发展,促进职业教育和就业岗位零距离对接。建设南邮通达学院二期工程、扬州技师学院新校区。围绕打造“健康中国的扬州样本”,努力提供全方位全周期健康服务。开工建设市公共卫生中心、市妇女儿童医院。加快推进分级诊疗,基层医疗卫生机构标准化建设达标率95%以上。4家农村区域性医疗卫生中心创成二级医院。创建全国基层中医药工作先进市。开展重点品种食品抽检9000批次,让人民群众吃得更放心。

实施富民增收政策,完善就业社保体系。深入实施市“富民增收39条政策”。城乡劳动者职业技能培训3万人次,城镇新增就业5.5万人。实施全民创业行动计划,新增市级创业孵化基地10个。进一步强化社保扩面征缴工作,增强社会保险基金市级统筹调剂能力。按照“六统一”要求,完善城乡居民医疗保险制度。完善社会救助,将“救急难”范围扩展到因病因祸因灾支出型贫困家庭。支持市慈善总会、红十字会开展人道救助。严格执行房地产市场调控各项措施,全力保持房地产市场平稳健康发展。

突出共建共治共享,切实提高社会治理水平。深化网格化基层社会治理机制建设。继续实施社区建设行动计划,加快社区“两场两园两店”和邻里服务中心建设,推广城市社区全科社工服务模式。全市新建21个颐养示范社区。积极迎接“七五”普法中期考核。严格执行领导干部接访下访和包案化解信访突出问题制度,加强法律援助,健全涉法涉诉信访有效办理和有序退出机制。推进立体化信息化社会治安防控体系建设,严厉打击各类违法犯罪行为。做好非法集资、互联网金融等重点领域风险防范和处置。压实安全生产责任,加强交通运输、建筑施工、化工等重点行业领域安全生产治理。加强国防动员建设,强化后备力量改革,进一步做好双拥工作。

既尽力而为、又量力而行,继续办实办好民生实事。围绕“古城再提升、城区再完善、城乡全覆盖”目标,全面推进“厕所革命”,新增农村卫生改厕9000座,新改建城乡标准化卫生公厕53座、2A级以上旅游厕所40座。全市棚改基本建成1.6万套,整治老小区98.2万平方米,开展老小区多层住宅加装电梯试点。市区改造提升农贸市场8个,建设20个农贸市场快检示范站;实施公共租赁住房保障450户,供应限价商品住房300套,改造“城中村”15个、背街小巷36条,实施公房解危2.93万平方米;增扩社区停车泊位2943个。推进“四好农村路”建设,新改建农村公路380公里、农桥45座、农村机耕桥118座。改造农村危房856户。落实省中小学(幼儿园)助学政策,新招收宏志班20个。全市新增残疾人就业1000名,对1000名残疾儿童实施抢救性康复。对分散供养的特困人员,全面签订并落实关爱照料协议。

6、全面增强干事创业本领,努力建设务实高效法治清廉政府,提升服务高质量发展的能力和水平。

强化政治建设。牢固树立“四个意识”,增强“四个自信”,坚决维护以习近平同志为核心的党中央权威和集中统一领导,坚定执行党的政治路线,严格遵守政治纪律和政治规矩,开展“不忘初心、牢记使命”主题教育年活动,推动习近平新时代中国特色社会主义思想在扬州落地生根。

增强改革本领。持续深化“放管服”改革,争取市、县(市、区)整体开展相对集中行政许可权改革试点,推动“3550”“不见面审批”常态化机制化。加快建立现代财政预算和绩效管理制度,严格控减政府债务。推进农村土地“三权分置”改革,完善土地承包经营权流转机制。继续深化公立医院综合改革,建立与医疗服务成本相适应的价格动态调整机制。完成环保垂直管理改革任务,完善生态环境补偿制度,做好环境保护税征收改革工作。

提升服务效能。积极改进组织和领导经济工作方式,提升对经济运行分析研判、预警监测、有效校准的工作水平。深化统计管理体制改革,组织开展第四次经济普查。制定实施第五个服务企业“2号文件”。强化政策集成,推动领导走访服务常态化,精心精准帮助企业解决现实难题。践行“马上就办”,积极回应群众和企业关切,不断提升办事服务效率。健全和落实鼓励激励、容错纠错等机制,鼓励广大干部在敢于担当中创新实干、在勇于负责中服务发展。

推进依法行政。健全重大行政决策程序,推进法治政府建设。自觉接受市人大及其常委会的法律监督、工作监督和市政协的民主监督,做好各级政府重大决策出台前向本级人大报告工作,把协商民主贯穿政府履行职能全过程。完善政府法律顾问、公职律师制度。严格规范公正文明执法。加大政务公开力度。健全群众监督、舆论监督机制。

坚守廉政底线。突出问题导向,对确定的目标、制定的政策、作出的承诺,言出必行,一抓到底。延伸“三直接”关键环节操作规范,强化工程招投标、质量安全、资金拨付等风险防控,完善权力运行制约和监督机制。深入开展经济责任审计,加大政府投资工程、民生保障支出的审计力度。严格落实中央“八项规定”精神,坚决纠正“四风”,严控一般性支出,“五公”经费实现零增长。

各位代表!新时代开启新征程,新征程呼唤新作为。让我们更加紧密地团结在以习近平同志为核心的党中央周围,在中共扬州市委的坚强领导下,不忘初心,牢记使命,砥砺前行,为“迈上新台阶、建设新扬州”而继续努力奋斗!
